# Insights into population structure of East African sweetpotato cultivars from hybrid assembly of chloroplast genomes

**DOI:** 10.12688/gatesopenres.12856.2

**Published:** 2020-07-21

**Authors:** Chenxi Zhou, Tania Duarte, Rocio Silvestre, Genoveva Rossel, Robert O. M. Mwanga, Awais Khan, Andrew W. George, Zhangjun Fei, G. Craig Yencho, David Ellis, Lachlan J. M. Coin

**Affiliations:** 1 Institute for Molecular Bioscience, University of Queensland, St Lucia, Brisbane, QLD, 4072, Australia; 2 International Potato Center, P.O. Box 1558, Lima 12, Peru; 3 International Potato Center, P.O. Box 22274, Kampala, Uganda; 4 Plant Pathology and Plant-Microbe Biology Section, School of Integrative Plant Science, Cornell University, Geneva, NY, 14456, USA; 5 Data61, CSIRO, Ecosciences Precinct, Brisbane, QLD, 4102, Australia; 6 Boyce Thompson Institute, Cornell University, Ithaca, NY, 14853, USA; 7 Department of Horticulture, North Carolina State University, Raleigh, North Carolina, 27695, USA

**Keywords:** chloroplast, sweetpotato, genome assembly, Oxford Nanopore sequencing, Illumina sequencing, phylogenetic analysis, Convolvulaceae Ipomoea

## Abstract

**Background:** The chloroplast (cp) genome is an important resource for studying plant diversity and phylogeny. Assembly of the cp genomes from next-generation sequencing data is complicated by the presence of two large inverted repeats contained in the cp DNA.

**Methods:** We constructed a complete circular cp genome assembly for the hexaploid sweetpotato using extremely low coverage (<1×) Oxford Nanopore whole-genome sequencing (WGS) data coupled with Illumina sequencing data for polishing.

**Results:** The sweetpotato cp genome of 161,274 bp contains 152 genes, of which there are 96 protein coding genes, 8 rRNA genes and 48 tRNA genes. Using the cp genome assembly as a reference, we constructed complete cp genome assemblies for a further 17 sweetpotato cultivars from East Africa and an *I. triloba* line using Illumina WGS data. Analysis of the sweetpotato cp genomes demonstrated the presence of two distinct subpopulations in East Africa. Phylogenetic analysis of the cp genomes of the species from the Convolvulaceae *Ipomoea* section *Batatas* revealed that the most closely related diploid wild species of the hexaploid sweetpotato is *I. trifida*.

**Conclusions: **Nanopore long reads are helpful in construction of cp genome assemblies, especially in solving the two long inverted repeats. We are generally able to extract cp sequences from WGS data of sufficiently high coverage for assembly of cp genomes. The cp genomes can be used to investigate the population structure and the phylogenetic relationship for the sweetpotato.

## Introduction

The chloroplast (cp) genome has been widely used to study the phylogeography, molecular systematics and the population genetics for plants ^1, 2^. The chloroplast DNA (cpDNA) usually displays uniparental inheritance and represents a relatively high degree of conservation in genome structure and gene content ^2^. There are over 800 complete cp sequences available for a wide variety of plants from National Center for Biotechnology Information (NCBI) repository ranging in size from 107 to 218 Kb ^3^. The cp genomes usually contain 110–130 protein encoding genes (PEGs), about 30 transfer RNA (tRNA) genes and four ribosomal RNA (rRNA) genes, primarily participating in the process of photosynthesis ^3, 4^. The cpDNA typically forms a circular quadripartite structure with two inverted repeats (IRs), IRA and IRB, separated by one large single-copy section (LSC) and one small single-copy section (SSC) ^5^.

The first cpDNA was sequenced from tobacco ( *Nicotiana tabacum*) using the bacterial artificial chromosome (BAC) sequencing method in 1986 ^6^. The two IRs were cloned separately in order to distinguish between them. A plethora of cpDNA had since been sequenced with similar methods ^7– 9^. Besides BAC sequencing, an alternative strategy used to sequence cpDNA is whole-cp-genome amplification by rolling-circle amplification (RCA) technology ^10– 12^. However, both approaches require complicated library preparation.

The development of next-generation sequencing (NGS) technologies such as Illumina and Roche 454 facilitate faster and cheaper methods to sequence cp genomes ^13– 15^. The output of the NGS technologies is short reads of size up to a few hundred base pairs. It is difficult to assemble cp genome with short reads only, especially because of the two large IRs of tens of kilobase pairs. In order to solve this problem, a reference cp genome, normally from a related species, is usually used to anchor the contigs assembled from the short reads ^4, 16^. The long reads generated from the third-generation sequencing (TGS) technologies, such as the single-molecule real-time (SMRT) PacBio sequencing and Oxford Nanopore sequencing, can also be used to anchor the contigs and solve the repetitive regions. It is even possible to assemble cp genomes directly from long reads ^17^. However, as the sequencing error rate of the long reads from the TGS is typically higher than 10%, it is important to introduce an error correction step to guarantee an accurate genome assembly ^18^. The high-quality NGS short reads can be integrated for error correction to improve accuracy ^19, 20^.

The aforementioned methods to construct cp genomes from NGS or TGS data assume pure cpDNA were sequenced. More precisely, the cpDNA were isolated from the nuclear DNAs and other organelle DNAs before sequencing ^4, 13– 16^. However, whole-genome sequencing (WGS) data generated from NGS or TGS technologies always contains cp sequences at various levels determined by the tissue type and library preparation. Normally we are able to gain enough coverage of cp genome for assembly even from low coverage WGS data. There have since been several studies describing assembly of cp genomes from WGS data ^21– 27^. Extraction of cp sequences from the WGS data plays a key role in these methods. The most straightforward idea is to use a reference cp genome. The cp sequences could be extracted by examining the mapping results of the WGS data to the reference cp genome ^21, 22^. An alternative strategy relies upon the fact that there are many more copies of the cpDNA than the nuclear DNA and that from other organelles. The entire WGS data is assembled to construct contigs. Contigs that represent significantly higher coverages are treated as cp contigs ^23– 25^. NOVOPlasty adopted a seed-and-extend paradigm, where the seed could be a cp read sequence, a conserved gene or a cp genome from a related species ^26^. The start and the end of a given seed sequence are iteratively extended with reads that are overlapped with the seed until the circular genome is formed. Izan *et al.* proposed a K-mer frequency-based selection of cpDNA sequences from WGS data, which was integrated into a reference free cp genome assembler for non-model species ^27^.

Sweetpotato ( *Ipomoea batatas*) ranks among the ten most important food crops worldwide ^28^. The total annual production is more than 100 million metric tonnes grown on about 8.6 million hectares around the world in year 2016 ^29^. Understanding the sweetpotato genomes is of significant importance to achieve the full potential of the sweetpotato ^30^. Sweetpotato is a hexaploid (2n=6x=90) with genome size estimated to be between 2,200 to 3,000 Mb ^28^. Due to the complex genome structure, the availability of sweetpotato genomic resources is lacking. Under these circumstances, the cp genome provides researchers with an easy and efficient way to study sweetpotato ^4, 16, 31, 32^. A number of cp genomes from the genus *Ipomoea* have been sequenced ^4, 16, 33, 34^. Most of them are diploid wild relatives of the sweetpotato. The genome size is around 161 Kb, and the structure represents a standard quadripartite circular with a LSC of 87 Kb, a SSC of 12 Kb and two IRs of 31 Kb ^4^. The cp genomes were mainly used to perform phylogenetic analyses ^4, 16, 34^.

In the present study, we constructed a complete cp genome assembly for the hexaploid sweetpotato cultivar Tanzania ^35^ using long reads produced by the Oxford Nanopore sequencing technology. Despite the <1× genome coverage, we obtained approximately 270× data coverage for the cp genome. Illumina sequencing data was integrated to improve the accuracy of the genome assembly. Using the Tanzania cp genome assembly as a reference, we constructed 19 cp genomes for a further 17 sweetpotato cultivars (including a duplicate for one cultivar) and an *I. triloba* line from paired-end whole genome Illumina sequence data. The assembled sweetpotato cp genomes were combined to perform phylogenetic analysis to investigate the population structure of 18 East African sweetpotato cultivars. Putting together the assembled cp genomes and nine publicly available cp genomes of the sweetpotato and its wild relatives, we performed a phylogenetic analysis to investigate the phylogenetic relationship for species in Convolvulaceae *Ipomoea* section *Batatas*.

## Results

### Extraction of cp genome sequence from whole genome sequencing data

We generated high-coverage (60×) 150 bp paired-end Illumina WGS data, and low-coverage (<1×) Oxford Nanopore WGS data on a single cultivar, referred to as Tanzania ^35^ (Methods). The cultivar Tanzania was used as one of the parents to develop an F1 outcrossing mapping population (B×T) in the Genomic Tools for Sweetpotato (GT4SP) Improvement Project ^30^. Approximately 162,000 Nanopore reads and 1.46 billion Illumina reads were generated (Supplementary Table 1). A total of 6,710 Nanopore reads were identified for cp genome by mapping to 30 publicly available cp genomes of the species from the Convolvulaceae *Ipomoea* family ^4, 16, 33, 36^ (Methods, Supplementary Table 2). The total size is ~43.9 Mb, which represents ~270× data coverage for the cp genome. The longest read is ~30 Kb, and the average size is ~6.5 Kb (Supplementary Figure 1). We identified approximately 45 million Illumina reads for cp genome by mapping to the publicly available cp genomes summing to ~6.2 Gb, which were used for error correction for Nanopore reads and the genome assembly. The other parent for the B×T F1 outcrossing mapping population, Beauregard, was subject to whole genome sequencing at 60× coverage (Methods). A total of approximately 1.3 billion 150 bp Illumina reads were generated summing to ~164 Gb, of which approximately 52 million reads were identified as cp sequences with a total size of ~7.2 Gb (Supplementary Table 1). We performed Illumina WGS at 30× coverage for a further 16 sweetpotato cultivars—Wagabolige and New Kawogo ^35^, Ejumula and SPK004 ^37^, NASPOT 1 and NASPOT 5 ^38^, NASPOT 7 and NASPOT 10 O ^39^, NK259L and NASPOT 11 ^40^, Huarmeyano, Dimbuka-Bukulula and NASPOT 5/58 ^41^, Resisto ^42^, Magabali ^43^ and Mugande ^44^. These cultivars were used as the parental genotypes in the Mwanga Diversity Panel (MDP) which is an 8×8 diallele diversity mating panel constructed by the GT4SP project for genomic selection of the sweetpotato. While the great majority of these sweetpotato cultivars were from East African countries including Uganda and Kenya, Resisto was from USA and Huarmeyano was from Peru (Supplementary Table 3). We have duplicate samples for the cultivar NASPOT 10 O—one was from the screen-house while the other one was from the field. These two NASPOT 10 O samples were analysed separately in this research (Methods). On average, a total of approximately 75 million 251 bp reads were generated for each sample. The number and the total size of the cp reads extracted from the whole genome sequence data, on average, are ~4.4 million and ~1 Gb respectively for each sample (Supplementary Table 1).

We performed Illumina whole genome sequencing at 50× coverage for the *I. triloba* line, NCNSP-0323 ^30^ (Methods). The raw whole genome sequence data consists of approximately 196 million 150bp reads summing to ~29 Gb. We extracted approximately 13 million reads for the cp genome from the raw sequence data summing to ~2 Gb (Supplementary Table 1).

### Cp genome assembly for the sweetpotato cultivar Tanzania

**Figure 1.**
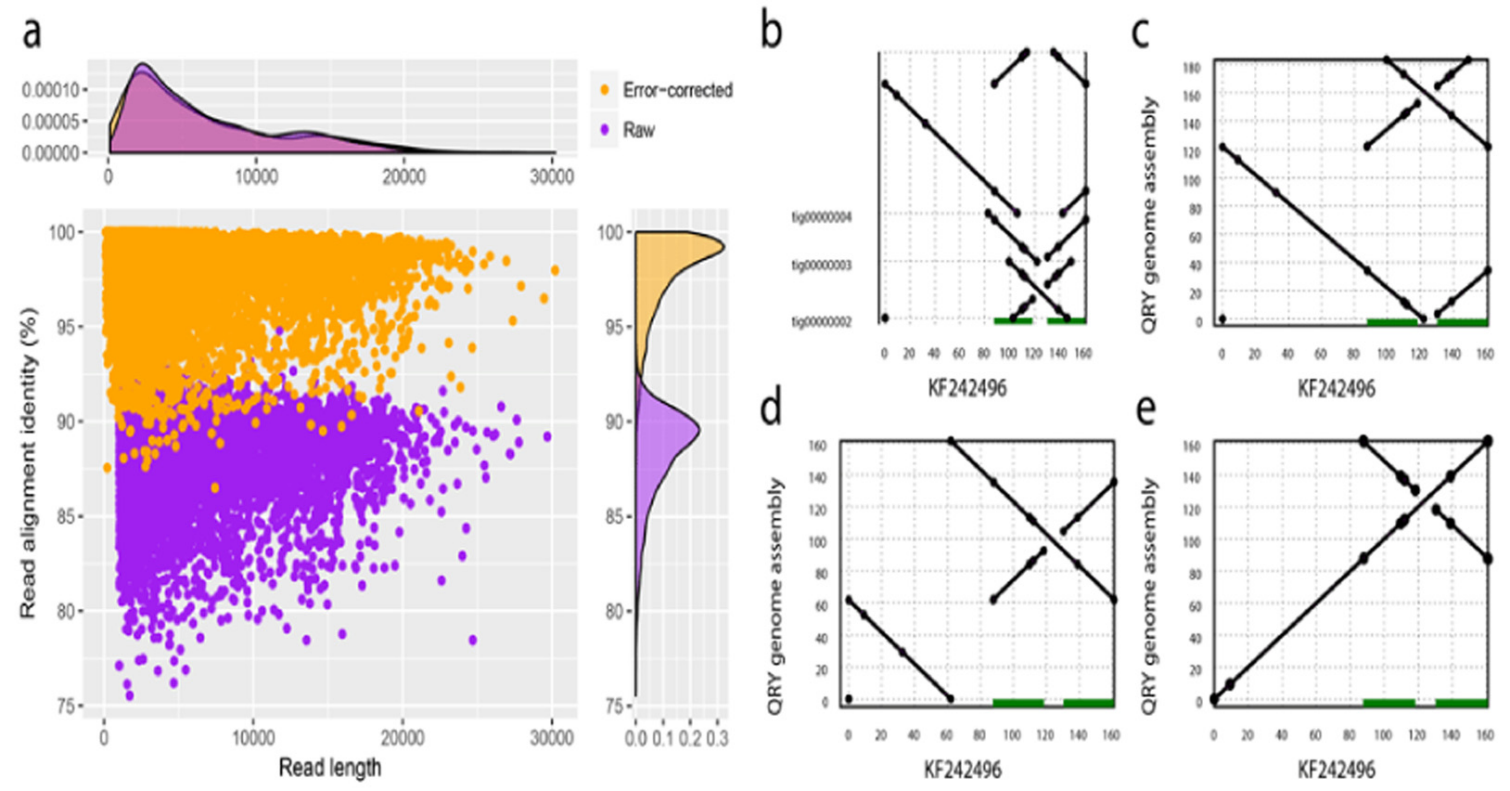
Assembly of the Tanzania chloroplast (cp) genome. ( **a**) Dot plot of the Nanopore read length versus the alignment identity to reference assembly. The read alignment identity is defined as *I* = *M/L*, where *M* is the total number of base pairs of the exact match and *L* is the size of the alignment span on the reference genome. The reference genome is the 30 cp genomes downloaded from the NCBI (Supplementary Table 2). The alignment was performed with BWA MEM ^45^. The alignment identities were calculated from the Cigar string. The purple and yellow represents before and after error correction with Illumina reads using Nanocorr ^20^, respectively. ( **b**) Dot plot of the reference cp genome versus the contigs produced by Canu ^17^. ( **c**) Dot plot of the reference cp genome versus the contigs produced by AMOS minimus ^46^ after merging Canu contigs. ( **d**) Dot plot of the reference cp genome versus the contigs produced by AMOS minimus after circularization. ( **e**) Dot plot of the reference cp genome versus the final cp genome assembly which was polished with Illumina reads using Pilon ^19^ and fixed the start at the LSC. For ( **b**– **e**), the cp genome assembly of the *I. trifida* was used as the reference (accession number REM 753, Genbank accession number KF242496) ^16^. The green bars on the *x*-axis indicate positions of the two IRs.

We combined the Nanopore long reads with Illumina short reads to construct a cp genome assembly for the sweetpotato cultivar Tanzania (Methods). After trimming off the low-quality bases, approximately 2.2 Gb Illumina sequence data remained which was used for error correction for the Nanopore reads with Nanocorr (Supplementary Table 1). A total of 70 low quality Nanopore reads were removed after error correction and the total size reduced to approximately 43.2 Mb ( Figure 1a), which was used to construct a draft genome assembly using Canu. The resulting genome assembly of approximately 218 Kb consists of three contigs of size 46 Kb, 39 Kb and 132 Kb, respectively. Compared to the published sweetpotato cp genome, the assembly is split at the boundaries of the two IRs ( Figure 1b). Utilizing the overlap information between the contigs, the AMOS minimus combined the three contigs and generated a single contig of ~183 Kb ( Figure 1c) (Methods). The contig contains a ~20 Kb redundancy at the ends which was removed after circularization ( Figure 1d). The circularized contig is ~161 Kb, and is highly collinear with the reference cp genome assembly ( Figure 1d). Application of Pilon further identified and corrected 42 single-nucleotide polymorphisms (SNPs) and small indels. To follow the paradigm of the published cp genomes, we restructured the genome assembly so that it starts from the LSC (Methods). The final genome assembly consists of a single circular contig of 161,274 bp ( Figure 1e).

### Cp genome assembly for the other 17 sweetpotato cultivars and the *I. triloba* line NCNSP-0323

The cp sequence data was subjected to quality control before assembled with SPAdes (Methods). After trimming off the low-quality regions, the total sizes of the sequence data of the 19 samples range from approximately 267 Mb to 2.67 Gb (Supplementary Table 1). The contigs generated from SPAdes for the 19 samples vary in numbers and sizes: the minimum number of contigs is 76 for the cultivar NASPOT 7, while the maximum number is 197 for the cultivar Beauregard; and the total sizes of the genome assemblies range from ~169 Kb (cultivars Ejumula and NASPOT 7) to ~229 Kb (cultivar NK259L) (Supplementary Table 4). The SPAdes contigs were then mapped to the Tanzania cp genome assembly for anchoring (Methods). The resulting genome assemblies for the 19 samples are very similar. The largest and the smallest genome assembly is 161,509bp and 161,198bp, derived from the cultivar NASPOT 5 and Beauregard, respectively (Supplementary Table 4).

### Molecular structure and gene content of the sweetpotato cp genome

**Figure 2.**
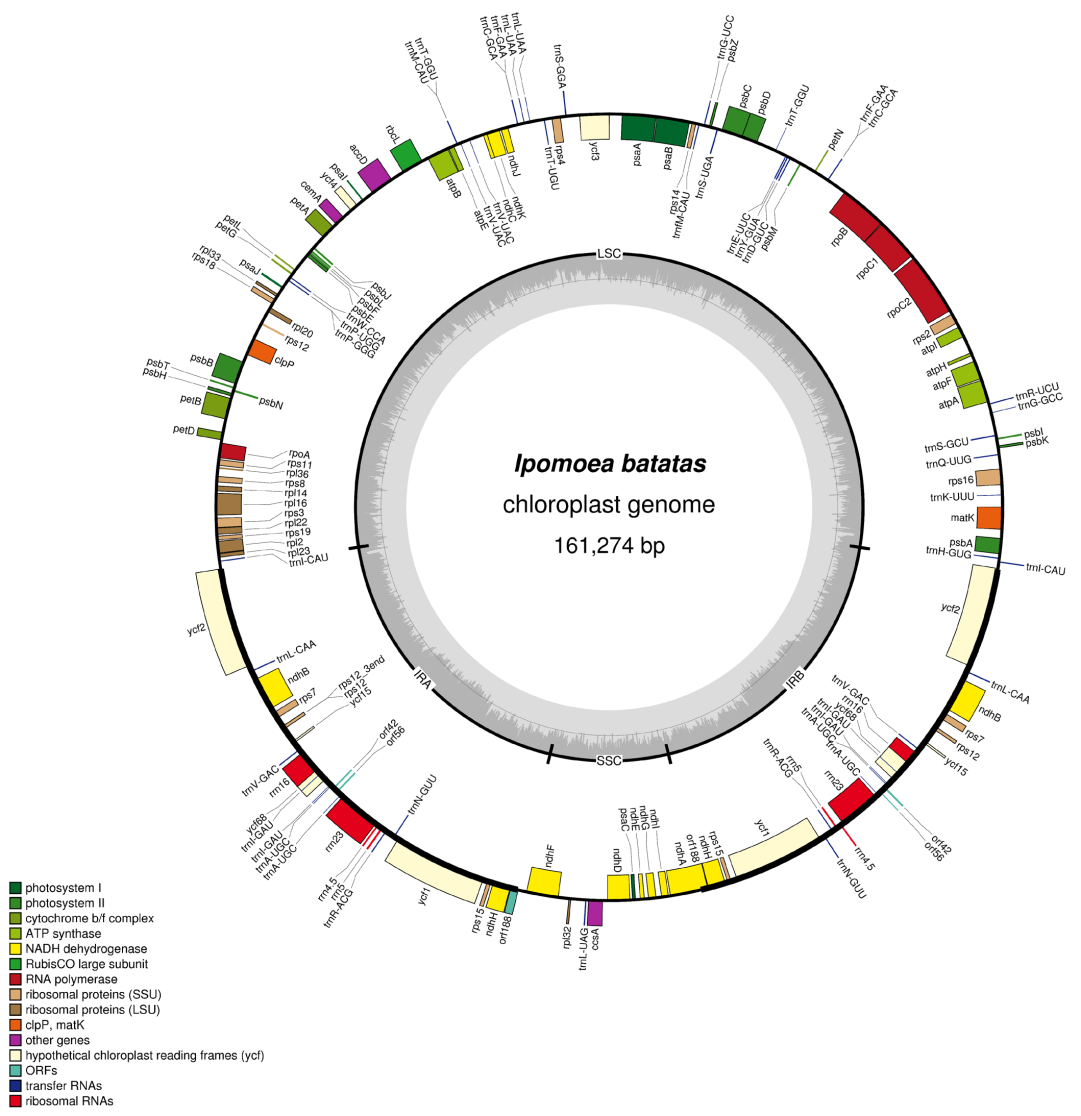
The chloroplast genome of the sweetpotato cultivar Tanzania. The preliminary annotations were produced by DOGMA ^48^. MUSCLE ^49^ was used to refine the annotations. The plot was generated with OGDRAW ^50^.

The gene annotation of the cp genome assembly of the sweetpotato cultivar Tanzania was generated with the web tool DOGMA and further refined with MUSCLE (Methods). The circular plot of the gene annotation is depicted in Figure 2. The sweetpotato cp genome represents a common circular structure with two IRs (IRA and IRB) separating one LSC and one SSC2. The size of the IRA, IRB, LSC and SSC is 30,874, 30,835, 87,489 and 12,076 bp, respectively. The overall GC content of the sweetpotato cp genome is 37.54%. The GC contents in different regions are highly variable. The two IRs represent significantly higher GC content than the single-copy regions: for the LSC and SSC, the GC content is 36.14% and 32.20%, respectively, whereas for the two IRs, the GC content is 40.57%. This is mainly caused by the high GC content ribosomal RNA genes in IR regions, including *rrn16*, *rrn23*, *rrn4.5* and *rrn5* ( Figure 2). We identified 152 genes in the cp genome of which there are 96 protein encoding genes (PEGs), eight rRNA genes and 48 tRNA genes. Table 1 shows a full list of the functional genes. As we can see, the genes can be divided into 16 functional systems. The number of single-copy and double-copy genes is 71 and 11, respectively, and there is one triple-copy gene ( *rps12*). The results are highly similar to what has been reported for the cultivar Xushu 18 cp genome ^4^; the only difference is that the *psbZ* gene is not found in the cultivar Xushu 18 cpDNA while the *ihbA* gene is not found in the cultivar Tanzania cpDNA. It should be noted that the double-copy gene *ycf1* was not reported for the cultivar Xushu 18 cp genome4, but this was actually a miss-annotation.

**Table 1.** List of annotated genes. The functional systems were adopted from the OGDRAW ^50^. Bracketed superscripts represent number of copies.

Functional system	Number	Gene list
Photosystem I	7	*psaA, psaB, psaC, psaI, psaJ, ycf3, ycf4*
Photosystem II	15	*psbA, psbB, psbC, psbD, psbE, psbF,* *psbH, psbI, psbJ, psbK, psbL, psbM,* *psbN, psbT, psbZ*
Cytochrome b/f complex	6	*petA, petB, petD, petG, petL, petN*
ATP synthase	6	*atpA, atpB, atpE, atpF, atpH, atpI*
NADH dehydrogenase	13	*ndhA, ndhB* ^[2]^, *ndhC, ndhD, ndhE, ndhF,* *ndhG,* *ndhH* ^[2]^ *, ndhI, ndhJ, ndhK*
RubisCO large subunit	1	*rbcL*
C-type cytochrome synthesis	1	*ccsA*
RNA polymerase	4	*rpoA, rpoB, rpoC1, rpoC2*
Ribosomal proteins (LSU)	9	*rpl2, rpl14, rpl16, rpl20, rpl22, rpl23,* *rpl32, rpl33, rpl36*
Ribosomal proteins (SSU)	16	*rps2, rps3, rps4, rps7* ^[2]^, *rps8, rps11, rps12* ^[3]^ *,* *rps14, rps15* ^[2]^ *, rps16, rps18, rps19*
Maturase K	1	*matK*
Acetyl-CoA carboxylase carboxyltransferase	1	*accD*
Clp protease proteolytic subunit	1	*clpP*
Chloroplast envelope membrane protein	1	*cemA*
ORFs	6	*orf188* ^[2]^, *orf42* ^[2]^, *orf56* ^[2]^
Hypothetical chloroplast RF	8	*ycf1* ^[2]^, *ycf15* ^[2]^, *ycf2* ^[2]^, *ycf68* ^[2]^

### Phylogenetic analysis of the sweetpotato cp genome

**Figure 3.**
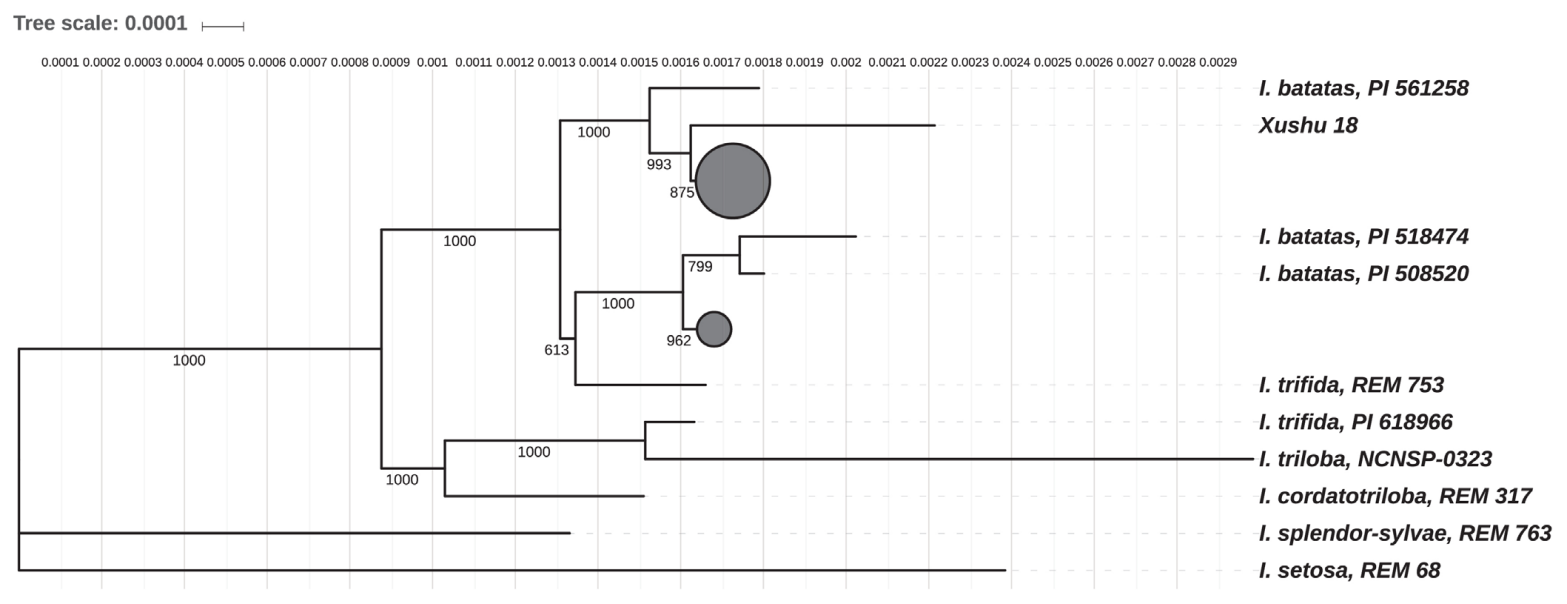
A phylogenetic tree of the Convolvulaceae *Ipomoea* section *Batatas* on the basis of chloroplast genomes. The numbers on the branches are bootstrap support values. The branches shorter than 2×10 ^-4^ substitutions per bp were collapsed resulting two clades consisting of 12 and 6 sweetpotato cultivars represented by a big and small solid circle respectively in the plot. The plot was generated with iTOL ^52^.

**Figure 4.**
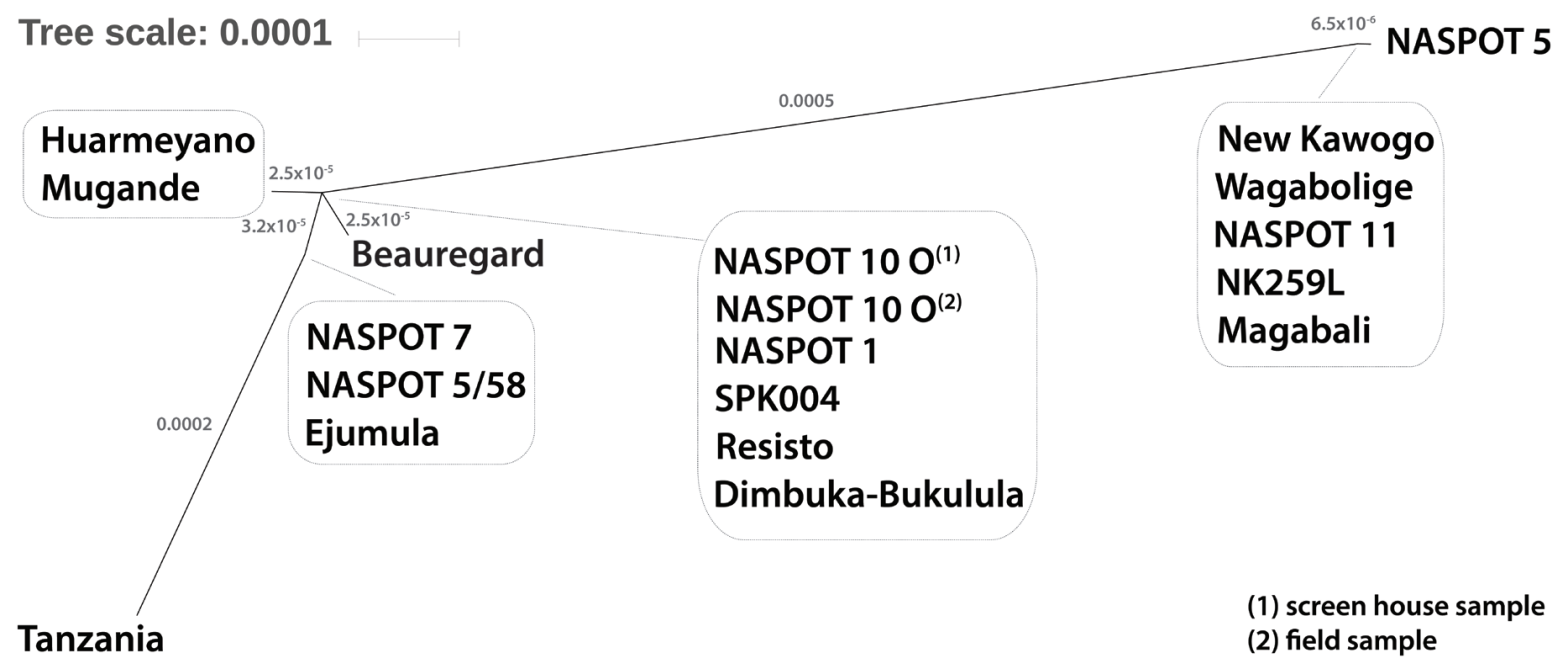
A phylogenetic tree of the East African sweetpotato cultivars used in the GT4SP project on the basis of chloroplast genomes. This is a fine-scale representation of the two clades in Figure 3. The numbers on the branches are branch lengths given in terms of substitutions per bp.

We performed a phylogenetic analysis for the Convolvulaceae *Ipomoea* section *Batatas* on the basis of the 19 cp genomes of the sweetpotato ( *I. batatas*) and the cp genome of the *I. triloba* line NCNSP-0323 assembled in this research, coupled with nine publicly available cp genomes, of which, four of them are for sweetpotato and two of them are for *I. trifida* and the other three are for *I. cordatotriloba*, *I. splendor-sylvae* and *I. setosa*, respectively ^4, 16^ (Supplementary Table 2). The resulted phylogenetic tree is depicted in Figure 3. The 18 sweetpotato cultivars used as the parental genotypes for mapping populations in the GT4SP project represent two distinct clades, consisting of 12 and six cultivars, respectively. Here, the length of any branch in a clade is no greater than 2×10 ^-4^ substitutions per bp. The detailed phylogenetic relationship of the 18 sweetpotato cultivars is shown in Figure 4. As we can see, the distance between the two clades is approximately 5×10 ^-4^ substitutions per bp. In the larger clades, the cultivar Tanzania represents a relatively larger distance (2×10 ^-4^ substitutions ber bp) compared to the other cultivars. The population structure discovered here is similar to the one revealed by using simple sequence repeat primers by David *et al*. with the exception of the classification of the sweetpotato cultivars NK259L, Resisto and Mugande ^47^ (Supplementary Table 3). For the publicly available sweetpotato cp genomes, PI 561258 and Xushu 18 are closely related to the larger clade, while PI 518474 and PI 508520 have a closer relationship with the smaller clade ( Figure 3). The diploid wild relative of the hexaploid sweetpotato, *I. trifida* (REM 753), displays a significantly closer relationship to the *I. batatas* compared to the other species in the Convolvulaceae *Ipomoea* section *Batatas*. The other *I. trifida* accession PI 618966, however, represents a much larger diversity to the *I. batatas* and shows a close relationship to the *I. triloba* line NCNSP-0323 assembled in this research. Interestingly, the accession PI 618966 was originally identified as *I. triloba* and was recently reidentified as *I. trifida* by the GRIN National Genetic Resources Program. Among the other three species in the Convolvulaceae *Ipomoea* section *Batatas*, the *I. cordatotriloba* (REM 317) is closely related to the *I. triloba* (NCNSP-0323) and *I. trifida* (PI 618966) and therefore displays much closer relationship to the *I. batatas* compared to the *I. splendor-sylvae* (REM 763) and *I. setosa* (REM 68).

## Discussion

The sweetpotato cp genome contains two ~31 Kb IRs which is very difficult for short-read *de novo* assemblers. There have been a few studies exploring the possibility to perform *de novo* assembly of organelle genomes with long reads especially with SMRT PacBio sequencing reads ^21, 26, 51^. In this study, we constructed a complete sweetpotato cp genome assembly using the long reads generated from Oxford Nanopore sequencing. Nanopore reads proved to be extremely powerful in assembling the cp genome, especially in solving long repetitive regions. The sweetpotato cp genome contains two ~31 Kb IRs, which is very difficult for short-read *de novo* assemblers. With the overlapping information from long reads; however, the problem can be easily resolved. Canu ^17^ provides a useful tool set for assembling Nanopore reads, which was used in this research. It is worth noting that although the average depth of coverage of the whole sweetpotato genome is less than 1×, we obtained enough coverage of the cp genome for assembly.

Although long reads are powerful in solving complex genome structures, the error-prone nature of the raw reads necessitates an extra error-correction step. Illumina reads have been widely used to assist long read error-correction ^19, 20^. The Illumina read-based correction could be performed either on the raw long reads before assembling ^20^ or on the draft genome assembly constructed from the raw reads ^19^. In the current study, we did both. Before assembling with Canu, the Nanopore reads were corrected with Illumina reads using Nanocorr ^20^. After assembling, the draft genome assembly was polished with Illumina reads using Pilon ^19^ (Methods). With several pipelines examined, we found that to perform error correction both before and after assembling is the best practice to construct the sweetpotato cp genome.

Assembling the cp genome from the short Illumina reads is challenging owing to the two large IRs. Since the structure of the cp genome is generally stable, reference genomes from the closely related species are usually used to perform reference-based assembling ^4, 16^. In this study, we used the genome assembly constructed from the Nanopore reads as reference to assemble cp genomes for a further 19 cp genomes including 17 sweetpotato cultivars (including a duplicate for one sample) and the *I. triloba* line NCNSP-0323. SPAdes ^53^ was used as the *de novo* short-read assembler. The contigs generated by SPAdes were fragmented as expected. Among the 19 genome assemblies, the minimum number of contigs was 76. As the two IRs are highly homologous, there was generally only one copy of repetitive regions being assembled. In order to solve this problem, for reference-based scaffolding, we reused some single-copy contigs from the two IR regions to construct complete cp genome assemblies.

The molecular structure and gene content of the cpDNA are relatively conserved in land plants ^2^. Many cpDNAs form a circular quadripartite structure with two IRs separated by one large and one small single-copy section ^2, 5^. All 20 cp genome assemblies constructed in this research represent this common structure. The size of the two IRs of the sweetpotato cpDNA is approximately 31 Kb each, and is much larger than the other plants such as potato ^10^, rice ^54^, wheat ^55^, and maize ^56^, of which the IRs are usually smaller than 26 Kb. This is highly likely due to gene losses in these species. By comparing the gene annotation of the sweetpotato cpDNA in this study ( Figure 2) to the potato cpDNA ^10^, we can see that, in the potato cpDNA, the boundary region of the IRA and SSC harbors a deletion of approximately 6Kb involved in the genes, *ycf1*, *rsp15* and *ndhH*. Meanwhile, these three genes are presented in the symmetric boundary region of the IRB and SSC, which explains why the size of the IRs of the potato cpDNA is approximately 6 Kb smaller than the sweetpotato cpDNA.

The cpDNA usually has uniparental inheritance and undergoes low rates of substitution and recombination, which makes it well suited for phylogenetic analysis. The cp genome has been widely used to perform phylogenetic or comparative analysis in previous studies ^2, 10, 16^. In this research, we used the complete cp genome assemblies to study the phylogenetic relationship of the 18 sweetpotato potato cultivars used as the parental genotypes for mapping populations in the GT4SP project, as well as the species from the Convolvulaceae *Ipomoea* section *Batatas*. The sweetpotato genotypes from the GT4SP project were classified into two distinct clusters, which guarantees the diversities of mapping populations derived from them. The phylogenetic analysis clearly revealed that the *I. trifida* is the most closely related diploid wild relatives to the hexaploid sweetpotato, *I. batatas*, which is consistent with conclusions from the previous studies ^32, 57^.

Almost all whole genome sequencing data contains cp sequences, from which we are usually able to obtain cp genome sequences of enough data coverage for *de novo* assembly. As we can see, all the cp genome assemblies described in this research were constructed using whole genome sequencing data. Given that the cp genome is an important resource for studying plant genomes and whole genome data has gradually become indispensable in modern genome projects, it will be a good practice to construct the cp genome assembly to gain a first insight into the plant genome we are trying to understand before moving to the complex nuclear genome.

## Methods

### Genome sequencing of the MDP parental genotypes

The 16 sweetpotato cultivars used as the parental genotypes for the MDP diversity panel were subjected to whole genome sequencing. These sweetpotato cultivars were collected from Uganda, Kenya, USA and Peru, and included Wagabolige, New Kawogo, Ejumula, SPK004, NASPOT 1, NASPOT 5, NASPOT 7, NASPOT 10 O, NK259L, NASPOT 11, Huarmeyano, Dimbuka-Bukulula, NASPOT 5/58, Resisto, Magabali and Mugande (Supplementary Table 3). Leaf tissue was ground to a fine powder using the FastPrep-24 ^TM^ 5G tissue homogenizer (MP Biomedicals, Santa Ana, California) and DNA extracted from the leaf tissues following published protocols with modifications ^58, 59^. Briefly, tissue was suspended in pre-warmed (65°C) CTAB buffer (200mM Tris-CL, 50mM EDTA, 2M NaCl, 2% CTAB and 3% *β*-mercapto-ethanol), mixed and heated at 65°C for 45 min prior to extraction with chloroform:isoamyl alcohol (24:1) and precipitated with sodium acetate and ethanol. Paired-end genomic libraries were prepared using the Illumina’s Genomic DNA Sample Preparation kit and sequenced on the Illumina HiSeq 2500 system with paired-end mode and read length of 251 bp (Illumina, San Diego, CA).

### 10x Genomics’ Chromium sequencing of the sweetpotato cultivar Tanzania and Beauregard

The genomic DNA of Tanzania and Beauregard were extracted using the method cetyltrimethyl ammonium bromide and purified with 1× Agencourt AMPure XP beads (Beckman Coulter), according to manufacturer’s instructions. Before the library preparation, 1.5 µg purified gDNA was size selected using the BluePippin instrument (Sage Science) with the 0.75% Agarose Dye free, Marker U1 High-pass 30–40 kb vs3 protocol followed by a purification step with 0.4× AMPure XP beads. The library preparations for these two samples were done following the Chromium ^TM^ Genome Reagent Kits user guide (CG00022, Rev C). In summary, 10 ng of sample DNA was used to generate Gel Bead-In-Emulsions (GEM) in the Chromium ^TM^ Controller (10× Genomics) followed by isothermal incubation, post GEM incubation cleanup and quality control (QC). Libraries were constructed with end-repair and A-tailing, adaptor ligation, post ligation cleanup using SPRIselect Reagent (Beckman Coulter, USA), sample index PCR, post PCR cleanup, and QC. We modified the protocol by increasing the number of PCR cycles to nice and adding 105 µl SPRIselect reagent for the Post Sample Index PCR Cleanup, which resulted in the recovery of shorter fragments than it was expected. The libraries were sequenced using the HiSeq X Ten platform (Illumina, San Diego, CA).

### Oxford Nanopore sequencing of the sweetpotato cultivar Tanzania

Before the MinION library preparation, 5.7 µg Tanzania pure DNA was size selected (start selection size: 8Kb) with the same protocol used in 10x Genomics’ Chromium sequencing. The size selected gDNA was purified with 1× AMPure XP beads. The resulting 950 ng of Tanzania gDNA was used in MinION sequencing library preparation with the SQK-LSK108 1D ligation Sequencing kit (May 2017 version). We modified the protocol as follows: 30 min incubation each end-repair step and adapter ligation; 10 min incubation at RT in the end-repair purification step; 0.7× AMPure XP beads used after adapters ligation and ELB buffer (Oxford Nanopore Technologies) warmed up at 50°C previously to use and incubation of the eluted solution at 50°C. A library of 348 ng was loaded into a FLO-MIN106 (R.9.4 version) flowcell used in a MK1B MinION. We run the 1D protocol in the MinKnow software (version 1.5.18) and we basecalled the raw data using Albacore (version 1.1.0).

### Cp genome sequence extraction

WGS data were aligned to 30 publicly available cp genome assemblies of the species from the *Ipomoea* family ^4, 16, 33, 36^ (Supplementary Table 2) to extract cp genome reads, using BWA MEM ^45^ (version 0.7.15). We used the option ‘-x *ont2d*’ for Nanopore reads, and default options for Illumina reads. For each Nanopore read, the alignment records with at least 500 bp sequence aligned were selected to calculate the total length of the alignment. A Nanopore read was considered as a cp sequence if at least 1 Kb and 80% of the read aligned. A similar strategy was employed for Illumina reads extraction. Both of the two reads of a read pair were required to be aligned. The minimum size of the alignment block was set to 100 bp.

### Cp genome assembly from Nanopore data

We used Nanocorr ^20^ (version 0.01) to perform error correction for Nanopore reads using the Illumina reads. In order to guarantee the quality of Illumina reads, Trimmomatic ^60^ (version 0.36) was used to remove the low quality regions. We imposed the quality score of each base pair to be no less than 20 and the length of the reads no less than 100. The corrected Nanopore reads were then used to construct a draft genome assembly with Canu ^17^ (version 1.5). As the resulting draft genome assembly contained more than one contig, AMOS minimus ^46^ (version 3.1.0) was used to remove the redundancy and concatenate contigs using the overlap information. The AMOS minimus was also used to circularize the contig. We aligned the Illumina reads to the circularized contig and corrected the SNPs and small indels with Pilon ^19^ (version 1.22). In order to follow the paradigms of the published cp genomes, we aligned the genome assembly to the published cp genomes with MUMMER ^61^ (version 3.23) to find homology regions, and let the genome assembly start from the LSC.

### Cp genome assembly from Illumina Hiseq data

The low quality regions of the extracted cp sequences were removed with Trimmomatic ^60^ (version 0.36). The minimum quality score of each base pair was set to 20 and the minimum length of the reads was set to 100. SPAdes ^53^ (version 3.10.1) was used to construct contigs from Illumina reads. We excluded the repeat resolve module from SPAdes and used the contigs before repeat resolution as it consistently missed one of the two IRs. The resulting genome assembly contains tens to hundreds of contigs. The size of the contigs ranged from several hundred base pairs to tens of kilobase pairs. Since we know the structure of cp genome is generally stable, the syntenic relationship was used for scaffolding. We mapped the SPAdes contigs to the genome assembly resulting from the Nanopore reads using BWA MEM ^45^. The alignments were used to order the contigs. The overlap information between the neighbouring contigs was used to concatenate them.

### Cp genome annotation

We used the web tool Dual Organellar GenoMe Annotator (DOGMA) ^48^ to generate the preliminarily gene annotations. For each particular gene, we used MUSCLE ^49^ (version 3.8.31) to align the genuine protein sequences of the gene gained from the NCBI GenBank to the genome assembly to decide the exact boundary positions. The web tool Organellar Genome DRAW (OGDRAW) ^50^ was used to generate the circular annotation plot of the genome assembly. The hypothetical cp open-reading frame *ycf1* was not identified by DOGMA initially. It was added to the annotation on the basis of the MUSCLE alignment results.

### Phylogenetic analysis

Phylogenetic analysis was performed on the 18 sweetpotato cultivars used as the parental genotypes for constructions of mapping populations in GT4SP project as well as the Convolvulaceae *Ipomoea* section *Batatas* including the cp genome assemblies constructed in this research and nine publicly available cp genome assemblies. MAFFT ^62^ (version 7.310) was employed to perform the multiple sequence alignment (MSA) for cp genomes. The phylogenetic structure was constructed with PhyML ^63^ (version 3.1). Branch certainty was evaluated with 1000 replications of bootstrap resampling. The phylogenetic tree depicted in this research was constructed with the web tool iTOL (version 4) ^52^.

## Data availability

### Underlying data

Nanopore and Illumina reads and the cp genome assemblies are deposited at NCBI BioProject repository, accession number PRJNA438020: http://identifiers.org/bioproject/PRJNA438020.

### Extended data

**Supplementary Figure 1. Size distribution of the Nanopore sequencing data of the total DNA.** https://doi.org/10.26188/12652034.v2 ^64^

**Supplementary Table 1. Statistics of the chloroplast (cp) sequencing data.** https://doi.org/10.26188/12652067.v1 ^65^

**Supplementary Table 2. List of the 30 publicly available Ipomoea chloroplast (cp) genomes in the NCBI repository.** https://doi.org/10.26188/12652079.v1 ^66^

**Supplementary Table 3. Description of the parental genotypes of the Mwanga Diversity Panel (MDP).** https://doi.org/10.26188/12652085.v1 ^67^

**Supplementary Table 4. Statistics of the chloroplast (cp) genome assemblies of the 18 sweetpotato cultivars and the *I. triloba* line NCNSP-0323.** https://doi.org/10.26188/12652094.v1 ^68^
