# Supplementary material for: Insights into population structure of East African sweetpotato cultivars from hybrid assembly of chloroplast genomes
Source: Gates Open Res. 2020 Jul 21;2:41. Originally published 2018 Sep 5. [Version 2] doi: 10.12688/gatesopenres.12856.2 (PMC7536352; doi:10.12688/gatesopenres.12856.2)
Supplement: Peer review history

# Peer review: Reviewer response for version 2

**DOI:** 10.21956/gatesopenres.14357.r29627

**Published:** 2020-10-07

**Authors:** Zongyun Li

**Affiliations:** 1 Institute of Integrative Plant Biology, School of Life Science, Jiangsu Normal University, Xuzhou, Jiangsu 221116, China; Jiangsu Key Laboratory of Phylogenomics and Comparative Genomics, Jiangsu Normal University, Jiangsu, China

Summary of the key results:

 The authors sequenced and assembled 19 cp genomes of sweetpotato cultivars and one wild species using Oxford Nanopore and Illumina sequencing. With the published data, the authors constructed a phylogeny tree and proposed that *I. trifida* is the most closely related diploid species of the sweetpotato. In addition, 18 sweetpotato cp genomes demonstrated the presence of the two distinct subpopulations in East Africa.

 Overall evaluation:

 This manuscript provided more information for sweetpotato genome resources which is very important for us to learn more about the genetic diversity, origin, and evolution of sweetpotato. According to the contents of results and discussion, some comments were listed as follows:

1. Park *et al.* (2018 ^1^) and Sun *et al.* (2019 ^2^) also published some *Ipomoea* cp genomes. It would be better to cite their works.
2. There are more genes annotated in this article than in other studies (Eserman *et al.*, 2014 ^3^; Yan *et al.*, 2015 ^4^; Park *et al.*, 2018 ^1^; Sun *et al.*, 2019 ^2^). Can the authors discuss the reasons? Which genes were not annotated in previous studies?
3. I thought one of the highlights of this paper was the assembly of 18 sweetpotato cp genomes. The authors demonstrated the presence of the two distinct subpopulations in East Africa using these cp genomes. However, no other more detailed analysis and discussion about these 18 cp genomes. Would it be better to add more detailed sequences analysis? For example, the factors which impact the genome size, some specific loci between the two subpopulation, etc.

 

 Overall, this manuscript is valuable for indexing.

Is the work clearly and accurately presented and does it cite the current literature?

Partly

If applicable, is the statistical analysis and its interpretation appropriate?

Yes

Are all the source data underlying the results available to ensure full reproducibility?

Yes

Is the study design appropriate and is the work technically sound?

Yes

Are the conclusions drawn adequately supported by the results?

Yes

Are sufficient details of methods and analysis provided to allow replication by others?

Yes

Reviewer Expertise:

NA

I confirm that I have read this submission and believe that I have an appropriate level of expertise to confirm that it is of an acceptable scientific standard.

## References

[1] : The Complete Chloroplast Genomes of Six Ipomoea Species and Indel Marker Development for the Discrimination of Authentic Pharbitidis Semen (Seeds of I. nil or I. purpurea). *Front Plant Sci*.2018;9: 10.3389/fpls.2018.00965 965 10.3389/fpls.2018.00965 30026751PMC6041466

[2] : A systematic comparison of eight new plastome sequences from Ipomoea L. *PeerJ*.2019;7: 10.7717/peerj.6563 e6563 10.7717/peerj.6563 30881765PMC6417408

[3] : Phylogenetics and diversification of morning glories (tribe Ipomoeeae, Convolvulaceae) based on whole plastome sequences. *Am J Bot*.2014;101(1) : 10.3732/ajb.1300207 92-103 10.3732/ajb.1300207 24375828

[4] : Analyses of the complete genome and gene expression of chloroplast of sweet potato [Ipomoea batata]. *PLoS One*.2015;10(4) : 10.1371/journal.pone.0124083 e0124083 10.1371/journal.pone.0124083 25874767PMC4398329

# Peer review: Reviewer response for version 2

**DOI:** 10.21956/gatesopenres.14357.r29625

**Published:** 2020-10-07

**Authors:** Qiang Li

**Affiliations:** 1 Xuzhou Institute of Agricultural Sciences in Jiangsu Xuhuai District, Key Laboratory for Biology and Genetic Breeding of Sweetpotato (Xuzhou), Ministry of Agriculture, Sweet Potato Research Institute (SPRI), Chinese Academy of Agricultural Sciences, Xuzhou, China

Overall evaluation:

 I think that the manuscript is acceptable. It gives us a new method to insight into the population structure of plants, especially sweetpotato with complicated genome. However, I have a number of suggestions to the article.

  

1. Materials:
   
  1. Just the author’s response to Prof. Aureliano Bombarely, "the primary focus of this study was to investigate the population structure of the East African sweetpotato cultivars used in the GT4SP project", 16 cultivars for MDP and 2 cultivars (Tanzania and Beauregard) for constructing F1 mapping population are suitable for this research. The *I. triloba* was selected. You can give us reasons why you chose this wild species, not *I. trifida*, or other wild relatives.
  2. The authors assembled cp genomes 2 times of cultivar NASPOT 10 O - one was from the screen-house while the other one was from the field. What was the aim? You can give us more detail about the difference or consistency between them.
  3. There are total 20 samples, including 19 samples from 18 sweetpotato cultivars (2 samples of the cultivar NASPOT 10 O), and 1 sample of wild relative. I always confuse the number in the article, cultivars or samples.
2. Methods:
   
  1. "In the present study, we constructed a complete cp genome assembly for the hexaploid sweetpotato cultivar Tanzania using long reads produced by the Oxford Nanopore sequencing technology."  Is this a new method? Is it the first report? It is an efficient tool to assemble complicated genomes in my mind. It is an important part of this article. I suggest the method should be reflected in the title.
  2.  Give more detail in the discussion part about this new tool compared to other methods.
3. Results:
   
  1. "The sweetpotato cp genome of 161,274 bp contains 152 genes, of which there are 96 protein coding genes, 8 rRNA genes and 48 tRNAgenes..."  this is cp genome of Tanzania. There are a little difference among other cultivars.
  2. Suggest to compare the phylogenetic tree by using cp genome data and nuclear genome data, and to validate the method.
4. Others:
   
  1. "The only difference is that the *psbZ* gene is not found in the cultivar Xushu 18 cpDNA while the *ihbA* gene is not found in the cultivar Tanzania cpDNA. It should be noted that the double-copy gene *ycf1* was not reported for the cultivar Xushu 18 cp genome4, but this was actually a miss-annotation." The difference between Xushu 18 and Tanzania are *psbZ* and *ihbA*, why the *ycf1* was actually a miss-annotation. You should give more information about it.
  2. Just a suggestion from Dr. Yang, the important references should be added.

Is the work clearly and accurately presented and does it cite the current literature?

Partly

If applicable, is the statistical analysis and its interpretation appropriate?

Yes

Are all the source data underlying the results available to ensure full reproducibility?

Yes

Is the study design appropriate and is the work technically sound?

Yes

Are the conclusions drawn adequately supported by the results?

Yes

Are sufficient details of methods and analysis provided to allow replication by others?

Yes

Reviewer Expertise:

NA

I confirm that I have read this submission and believe that I have an appropriate level of expertise to confirm that it is of an acceptable scientific standard, however I have significant reservations, as outlined above.

# Peer review: Reviewer response for version 2

**DOI:** 10.21956/gatesopenres.14357.r29622

**Published:** 2020-10-05

**Authors:** Yuki Monden

**Affiliations:** 1 Graduate School of Environmental and Life Science, Okayama University, Okayama, Japan

I think this paper is well-written, and the authors have properly revised the manuscript by referring to the comments of the reviewer. This paper performed a complete circular cp genome assembly using NGS and TGS technologies in the hexaploid sweetpotato. Phylogenetic analysis using the cp genomes revealed that there are two distinct clusters of sweetpotato in East Africa and *I. trifida *is the most closely related diploid wild species to the I. batatas hexaploid sweetpotato. The results of this paper provide insights into the genetic relationships and the population structure of the species from the Convolvulaceae *Ipomoea *section Batatas. Besides, despite the complexity of the cp genomes by the presence of two large inverted repeats, this research demonstrates the possibility of building the cp genomes using extremely low coverage (<1x) Oxford Nanopore WGS data combined with Illumina short reads. Other comments are shown below.

  

1. Table 1:
  The copy number of rps12 gene should be three, but the bracketed superscript of this gene is two. Please make sure.
2. Phylogenetic analysis using nuclear genomes
  Is it possible to compare the results of phylogenetic analyses based on the cp genomes and the nuclear genomes using the same materials? I think such comparative analysis should provide new insight into evolutionary dynamics on cp and nuclear genomes of *Ipomoea *species. Do you have any plans for such work?

Is the work clearly and accurately presented and does it cite the current literature?

Yes

If applicable, is the statistical analysis and its interpretation appropriate?

Yes

Are all the source data underlying the results available to ensure full reproducibility?

Yes

Is the study design appropriate and is the work technically sound?

Yes

Are the conclusions drawn adequately supported by the results?

Yes

Are sufficient details of methods and analysis provided to allow replication by others?

Yes

Reviewer Expertise:

Plant genetics

I confirm that I have read this submission and believe that I have an appropriate level of expertise to confirm that it is of an acceptable scientific standard.

# Peer review: Reviewer response for version 1

**DOI:** 10.21956/gatesopenres.13938.r26743

**Published:** 2018-11-20

**Authors:** Aureliano Bombarely

**Affiliations:** 1 Department of Horticulture, Virginia Polytechnic Institute and State University, Blacksburg, VA, USA

**Summary of the key results**

 The manuscript titled “Insights into population structure of East African sweetpotato cultivars from hybrid assembly of chloroplast genomes” presents the chloroplast genome assembly and annotation of the sweet potato cultivar Tanzania and its comparison with seventeen other cultivars and the different species *Ipomoea triloba*. The final genome assembly of the Tanzania cultivar was 161,274 bp. No major findings were reported except for the deletion of the gene *psbZ* in the cultivar Xushu 18 and *ihbA* gene in the Tanzania. The phylogenetic analysis of these cultivars and nine publicly available *Ipomoea* chloroplast pointed that the diploid *I. trifida* species is close related to the hexaploid sweet potato ( *I. batatas*) than other *Ipomoea* species.

**Overall evaluation**

 This manuscript presents the assembly and analysis of the chloroplast genome of the sweet potato cultivar Tanzania. The *I. batatas* chloroplast genome was already published in 2015 by Yan et. al. (PLoS One 10:4) so the novelty of the results presented in this manuscript are limited from the point of new of a “new” chloroplast genome. The use of ON for the sequencing of the Tanzania cultivar and the addition of the resequencing data of seventeen other cultivars potentially could add some interesting findings. Nevertheless, the analysis that the authors performed failed in the development of attractive results. Personally, I would propose several analysis that it may help to increase the impact of the manuscript:

- Improved phylogenetic analysis partitioning the alignments per gene (or in bins) and using a Bayesian framework (e.g. BEAST). The phylogenetic analysis could include the dating of the divergency time of the different taxa.
- Nuclear gene mining. One of the most interesting questions about the polyploids is the origin of those. The use of the resequencing data could potentially derived in the mining of nuclear copy single gene that could help to elucidate a different evolutionary trajectory for these accessions. Additionally, it is interesting the result in which some genes are missing. Maybe they have been transferred to the nuclear genome. It could be interesting that hypothesis.
- Positive selection. Each of the chloroplast nuclear genes could be tested for positive selection using PAML and the Ks/Kn ratio.

 

 In terms of the manuscript organization and writing, I found confusing some parts. For example, the material and methods are not aligned with the results presented in the manuscript. For example, the section “Extraction of cp genome sequence from whole genome sequencing data” describe the chloroplast data mining from ON and Illumina for the Tanzania accession and then for the Beauregard accession, but the material and methods also describe the use of 10X Genomics Chromium that I am not sure where it comes from. Do the authors used 10X Genomics also? Probably for the genome assembly, the comparison of the Canu assembly with Organelle_PBA (Soorni et al. 2017) could be interesting, to see if the authors obtain only one contig representing the whole chloroplast.

 Overall, I think that the manuscript is okay, but there are some space for improvement in the structure of the manuscript and in the results that are presented.

Is the work clearly and accurately presented and does it cite the current literature?

Partly

If applicable, is the statistical analysis and its interpretation appropriate?

Yes

Are all the source data underlying the results available to ensure full reproducibility?

Yes

Is the study design appropriate and is the work technically sound?

Yes

Are the conclusions drawn adequately supported by the results?

Yes

Are sufficient details of methods and analysis provided to allow replication by others?

Yes

Reviewer Expertise:

NA

I confirm that I have read this submission and believe that I have an appropriate level of expertise to confirm that it is of an acceptable scientific standard, however I have significant reservations, as outlined above.

# Response

**Published:** 2020-07-12

**Authors:** Lachlan Coin

We thank the reviewer for their thoughtful review of  our manuscript. 

**Reviewers comment** 1. This manuscript presents the assembly and analysis of the chloroplast genome of the sweet potato cultivar *Tanzania*. The I. *batatas* chloroplast genome was already published in 2015 by Yan *et. al.* ( *PLoS* One 10:4) so the novelty of the results presented in this manuscript are limited from the point of new of a “new” chloroplast genome. The use of ON for the sequencing of the *Tanzania* cultivar and the addition of the resequencing data of seventeen other cultivars potentially could add some interesting findings. Nevertheless, the analysis that the authors performed failed in the development of attractive results. Personally, I would propose several analysis that it may help to increase the impact of the manuscript:

- Improved phylogenetic analysis partitioning the alignments per gene (or in bins) and using a Bayesian framework (e.g. BEAST). The phylogenetic analysis could include the dating of the divergency time of the different taxa.
- Nuclear gene mining. One of the most interesting questions about the polyploids is the origin of those. The use of the resequencing data could potentially derived in the mining of nuclear copy single gene that could help to elucidate a different evolutionary trajectory for these accessions. Additionally, it is interesting the result in which some genes are missing. Maybe they have been transferred to the nuclear genome. It could be interesting that hypothesis.
- Positive selection. Each of the chloroplast nuclear genes could be tested for positive selection using PAML and the Ks/Kn ratio.

**Response: **The primary focus of this study was to investigate the population structure of the East African sweetpotato cultivars used in the GT4SP project. We strongly agree that these suggested analyses will largely increase the impact of the manuscript. However, it is difficult to integrate these suggested analyses in the current study within the constraints of the data. We will include these suggestions in the future directions of the project.  

**Reviewers Comment: ** In terms of the manuscript organization and writing, I found confusing some parts. For example, the material and methods are not aligned with the results presented in the manuscript. For example, the section “Extraction of cp genome sequence from whole genome sequencing data” describe the chloroplast data mining from ON and Illumina for the *Tanzania* accession and then for the *Beauregard* accession, but the material and methods also describe the use of 10X Genomics Chromium that I am not sure where it comes from. Do the authors used 10X Genomics also? Probably for the genome assembly, the comparison of the Canu assembly with Organelle_PBA (Soorni et al. 2017) could be interesting, to see if the authors obtain only one contig representing the whole chloroplast.

**Response: **We have amended the mansucript to adddress these concerns.   10X Genomics was indeed used to perform the whole genome sequencing for the sweetpotato accessions *Tanzania* and *Beauregard*. However, the linked reads information was not utilized in construction of the cp genome assemblies for them. Instead, the sequence data was simply used as paired-end reads to create contigs. The contigs were then used to construct whole cp genome assembly with a cp reference genome.

# Peer review: Reviewer response for version 1

**DOI:** 10.21956/gatesopenres.13938.r26651

**Published:** 2018-09-13

**Authors:** Jun Yang, Mengxiao Yan

**Affiliations:** 1 State Key Laboratory of Plant Molecular Genetics, CAS Center for Excellence in Molecular Plant Sciences, Shanghai Institute of Plant Physiology and Ecology, Chinese Academy of Sciences, Shanghai, China; 2 Shanghai Key Laboratory of Plant Functional Genomics and Resources, Shanghai Chenshan Plant Science Research Center (SCPSRC), Shanghai Chenshan Botanical Garden, Chinese Academy of Sciences, Shanghai, China

Zhou *et al*. sequenced 16 sweet potato cultivars in GT4SP project supported by Bill & Melinda Gates Foundation. Here authors presented only partial data about chloroplast (cp) genome assemblies.

 I found this study will be a good supplementary work of previous publication in Current Biology ^1^. Due to the lack of awareness of this publication, the claims in the manuscript are incorrect and need to be revised.

 In this case, the finding of this study about two distinct cp subpopulations in East Africa cultivars is reasonable.

 Other comments

- “In recent years, the development of next-generation sequencing (NGS) technologies such as Illumina and Roche 454 facilitate faster and cheaper methods to sequence cp genomes ^13–15^.”
  To my knowledge, the Roche 454 already left the market.
- “By examining the mapping results of the WGS data, we are able to extract cp sequences ^21,22^.”
  We? Who are we?
- “Sweetpotato is a hexaploid (2n=6x=90) with genome size estimated to be between 2,200 to 3,000 Mb ^28^.”
  How about the C-values?
- “Due to the complex genome structure, the availability of sweetpotato genomic resources is lacking.”
  We do have a published genome, right?
- “A number of cp genomes from the *Ipomoea* family have been sequenced ^16,33^.”
  Dose *Ipomoea* family mean genus *Ipomoea*? Or genus *Ipomoea* Series *Batatas*?
- “Most of them are diploid wild relatives of the sweetpotato. To the best of our knowledge, to date, four cp genomes have been completely sequenced for the hexaploid sweetpotato ^4,16^; the genome size is around 161 Kb, and the structure represents a standard quadripartite circular with a LSC of 87 Kb, a SSC of 12 Kb and two IRs of 31 Kb ^4^. The cp genomes were mainly used to perform phylogenetic analyses ^4,16^.”
  The mentioned Current Biology paper has provided hundreds of cp genome sequences of sweet potato and its wild relatives.
- “The circularized contig is ~161 Kb, and is highly collinear with the published sweetpotato cp genome assembly (Figure 1d).”
  In Figure 1d, it is an *I. trifida* cp genome, not a published sweet potato cp genome.
- “The sweetpotato cp genome represents a common circular structure with two IRs (IRA and IRB) separating one LSC and one SSC2.”
  Where does the 2 in SSC2 come from? Convert into right format if it is a citation.
- “The red dots represent SNPs between the two cp genomes. The green bars on the x-axis indicate positions of the two IRs”
  No red dots there, only black dots.
- “It should be noted that the doublecopy gene ycf1 was not reported for the cultivar Xushu 18 cp genome4”
  Convert into right format if it is a citation.
- “Interestingly, the accession PI 618966 was originally identified as *I. triloba* and was recently reidentified as *I. trifida* by the GRIN National Genetic Resources Program.”
  The identification of PI 618966 needs to be checked carefully. All individuals of *I. trifida* formed a monophyletic clade closely related to *I. batatas* according to Current Biology paper. As the progenitor of sweet potato, it's quite strange that *I. trifida* is much closer to other species in Series *Batatas* than *I. batatas*.
- Figure 3 & 4
  It will be much clear to add the tip labels rather than collapsed clades on the tree. Figure 4 will be no more informative in this case.
  If the tree is not that complicated, it is not suggested to collapse the two clades. Since information about the relationship between within-clade sample and out-clade sample is not visible when one collapse clade. This information will not be illustrated in Figure 4. Clades can be labeled in different colors if one wants to highlight the clades. 
  Furthermore, it is not clear to me which place each sample nested on in Figure 4.
- “In this study, we used the genome assembly constructed from the Nanopore reads as reference to assemble cp genomes for a further 19 cp genomes including…”
  Misleading sentence, authors do rely on published cp genome rather than *de novo* Nanopore assembly.
- “In order to solve this problem, for reference-based scaffolding, we reused some single-copy contigs from the two IR regions to construct complete cp genome assemblies.”
  In which cultivar(s), did author investigate the influence on the tree structure?

I agree the population structure of East African sweet potato cultivars is important for GT4SP project. Also obviously, the data organization and visualization could be largely improved to meet the indexing standards.

Is the work clearly and accurately presented and does it cite the current literature?

Partly

If applicable, is the statistical analysis and its interpretation appropriate?

Yes

Are all the source data underlying the results available to ensure full reproducibility?

Yes

Is the study design appropriate and is the work technically sound?

Yes

Are the conclusions drawn adequately supported by the results?

Yes

Are sufficient details of methods and analysis provided to allow replication by others?

Yes

Reviewer Expertise:

Plant genetics

We confirm that we have read this submission and believe that we have an appropriate level of expertise to confirm that it is of an acceptable scientific standard, however we have significant reservations, as outlined above.

## References

[1] : Reconciling Conflicting Phylogenies in the Origin of Sweet Potato and Dispersal to Polynesia. *Curr Biol*.2018;28(8) : 10.1016/j.cub.2018.03.020 1246-1256.e12 10.1016/j.cub.2018.03.020 29657119

# Response

**Published:** 2020-07-12

**Authors:** Lachlan Coin

We thank the reviewer for their thoughtful review **.**

**Comment **1. I found this study will be a good supplementary work of previous publication in Current Biology (Mu, Pablo, *et al.*, 2018). Due to the lack of awareness of this publication, the claims in the manuscript are incorrect and need to be revised.

**Response: **This publication was cited in the revised version. The incorrect claim was revised (see reviewer’s comment 7).

**Comment **2. “In recent years, the development of next-generation sequencing (NGS) technologies such as Illumina and Roche 454 facilitate faster and cheaper methods to sequence cp genomes ^13–15^.”

To my knowledge, the Roche 454 already left the market.

**Response: **In the revised version, “In recent years” was deleted to make it more precise.

**Comment **3. “By examining the mapping results of the WGS data, we are able to extract cp sequences ^21,22^.”

We? Who are we?

**Response: **In the revised version, this sentence was rewritten to “The cp sequences could be extracted by examining the mapping results of the WGS data to the reference cp genome ^21, 22^.”

**Comment **4. “Sweetpotato is a hexaploid (2n=6x=90) with genome size estimated to be between 2,200 to 3,000 Mb ^28^.”

How about the C-values?

**Response: **The nuclear genome size is not the key point of this study. The C-value was not investigated.

**Comment **5. “Due to the complex genome structure, the availability of sweetpotato genomic resources is lacking.”

We do have a published genome, right?

**Response: **Even though there is a sweetpotato reference genome published recently (Yang, Jun, *et al.*, 2017), we think the availability of the sweetpotato genome resources is still lacking.  

**Comment **6. “A number of cp genomes from the *Ipomoea* family have been sequenced ^16,33^.”

Dose *Ipomoea* family mean genus *Ipomoea*? Or genus *Ipomoea* Series *Batatas*?

**Response:** “ *Ipomoea* family” means “genus *Ipomoea*”. This was made clear in the revised version.

**Comment **7. “Most of them are diploid wild relatives of the sweetpotato. To the best of our knowledge, to date, four cp genomes have been completely sequenced for the hexaploid sweetpotato ^4, 16^; the genome size is around 161 Kb, and the structure represents a standard quadripartite circular with a LSC of 87 Kb, a SSC of 12 Kb and two IRs of 31 Kb ^4^. The cp genomes were mainly used to perform phylogenetic analyses ^4, 16^. ”

The mentioned Current Biology paper has provided hundreds of cp genome sequences of sweet potato and its wild relatives.

**Response: **The claim “To the best of our knowledge, to date, four cp genomes have been completely sequenced for the hexaploid sweetpotato ^4, 16^;” was removed in the revised version.

**Comment **8. “The circularized contig is ~161 Kb, and is highly collinear with the published sweetpotato cp genome assembly (Figure 1d).”

In Figure 1d, it is an I. trifida cp genome, not a published sweet potato cp genome.

**Response: **This sentence was corrected as “The circularized contig is ~161 Kb, and is highly collinear with the reference cp genome assembly (Figure 1d).”

**Comment **9. “The sweetpotato cp genome represents a common circular structure with two IRs (IRA and IRB) separating one LSC and one SSC2.”

Where does the 2 in SSC2 come from? Convert into right format if it is a citation.

**Response:** This was a citation. It was corrected in the revised version.

**Comment **10. “The red dots represent SNPs between the two cp genomes. The green bars on the x-axis indicate positions of the two IRs”

No red dots there, only black dots.

**Response: **The red dots are hard to see due to the resolution of the image. Since the SNPs are not important in this genome assembly section and are further discussed in the phylogenetic analysis section, the SNPs (red dots) were removed from the figure 1d in the revised version.

**Comment **11. “It should be noted that the doublecopy gene ycf1 was not reported for the cultivar Xushu 18 cp genome4”

Convert into right format if it is a citation.

**Response:** This was a citation. It was corrected in the revised version.

**Comment **12. “Interestingly, the accession PI 618966 was originally identified as I. *triloba* and was recently reidentified as I. *trifida* by the GRIN National Genetic Resources Program.”

The identification of PI 618966 needs to be checked carefully. All individuals of I. *trifida* formed a monophyletic clade closely related to I. *batatas* according to Current Biology paper. As the progenitor of sweet potato, it's quite strange that I. *trifida* is much closer to other species in Series *Batatas* than I. *batatas*.

**Response: **We agree with the reviewer that the identification of PI 618966 needs to be checked carefully. According to the phylogenetic structure identified in this study (Fig. 3), PI 618966 has a closer phylogenetic relationship to I. *triloba *instead of I. *trifida*. However, it was recently reidentified as I. *trifida* by the GRIN National Genetic Resources Program. A further study is required to fully clarify this.

**Comment **13. Figure 3 & 4

It will be much clear to add the tip labels rather than collapsed clades on the tree. Figure 4 will be no more informative in this case.

If the tree is not that complicated, it is not suggested to collapse the two clades. Since information about the relationship between within-clade sample and out-clade sample is not visible when one collapse clade. This information will not be illustrated in Figure 4. Clades can be labeled in different colors if one wants to highlight the clades.

Furthermore, it is not clear to me which place each sample nested on in Figure 4.

**Response: **The two sweetpotato clades showed in Figure 3 were collapsed since the phylogenetic distances are too small. It will be impossible to see the detail phylogenetic structure of the two East African sweetpotato subpopulations if incorporate Figure 4 into Figure 3.

**Comment **14. “In this study, we used the genome assembly constructed from the Nanopore reads as reference to assemble cp genomes for a further 19 cp genomes including…”

Misleading sentence, authors do rely on published cp genome rather than *de novo* Nanopore assembly.

**Response: **The cp genome assembly of the sweetpotato cultivar *Tanzania* was constructed from the Nanopore reads coupled with a published cp reference genome. The cp genome assembly of the sweetpotato cultivar *Tanzania *from the Nanopore reads was then used as reference to constructed cp genome assemblies for a further 19 cp genomes.

**Comment **15. “In order to solve this problem, for reference-based scaffolding, we reused some single-copy contigs from the two IR regions to construct complete cp genome assemblies.”

In which cultivar(s), did author investigate the influence on the tree structure?

**Response: **Single-copy contigs were reused in construction of cp genome assembly for all cultivars. The genome assembler SPAdes collapsed the contigs from the two IR regions as they are almost identical. In order to construct the whole cp genome, the contigs from two IR regions need to be reused. This has no influence on the tree structure.
